# S07 How do sports clubs contribute to health? From theory to interventions

**DOI:** 10.1093/eurpub/ckac093.033

**Published:** 2022-08-29

**Authors:** Aurélie Van Hoye, Susanna Geidne, Katja Rinta-Antila, Kevin Gavin, Linda Ooms, Stacey Johnson, Jan Seghers, Sami Kokko

**Affiliations:** 1 APEMAC, University of Lorraine, Nancy, France; 2 Sport Department, Örebro University, örebro, Sweden; 3 Faculty of Sport and Health Sciences, University of Jyväskylä, Jyvaskyla, Finland; 4 Sport Department, Athlone Institute of Technology, Athlone, Ireland; 5 Mulier Instituut, Utrecht, The Netherlands; 6 Université Côte d'Azur, LAMHESS, France; 7 Physical Activity, sport and health research group, Katholieke Universiteit Leuven, Leuven, Belgium

**Keywords:** health promotion, sport clubs, settings-based approach

## Abstract

The symposium presents last findings on health promotion interventions in sports clubs. After a short introduction about the health promoting sports clubs (HPSC), five presentations (France, Sweden, Ireland, Finland and Netherlands) will reflect upon how sports clubs can be health promoting: in theory, from youth perspectives, by increasing physical activity level as outcome or enhancing sustainability of interventions, before opening the discussion with academic experts. Presentation 1 describes an iterative international process, implicating three groups (French sport students, French and Swedish experts) to create an intervention theory, based on the HPSC model. Presentation 2 focuses on a cross-sectional study investigating 123 Swedish youth's representation of sports clubs' role towards health promotion, identifying social dimension, environment, coaches, amount and ambition of practice as key factors. Presentation 3 is a longitudinal study among 366 adolescents, followed from age 15 to age 19, questioning the participation to organised sport practice and their orientation (leisure or competitive). Results have shown that by the age of 19, 33% of boys and 43% of girls have dropped out of organised sport, where 45% of boys and 26% of girls continued participation. Adolescents with a competitive goal orientation were more likely to continue participation. Presentation 4 is a longitudinal study among 131 youth measuring objective physical activity before and in the middle of a sport season. Principal results showed a significant change across time point, as well as differences between gender (a decrease in moderate to vigorous physical activity during games for boys and an increase for girls). Presentation 5 examined factors that influenced the sustainability of 14 Dutch sporting program aimed at increasing physical activity among inactive people 6.5 years after their implementation. Interviews with representatives of Dutch National Sports Federations and sports clubs helped to identify facilitating and impeding factors, like program adaptation, evaluation, financing and factors related to human resources. Question and Answer will be organised around the key ingredients and challenges facing the development of HPSC interventions, such as implementation of theoretical background, sport participants need consideration, complexity of outcomes evaluation of HPSC and program sustainability.

